# Obesity and outpatient rehabilitation using mobile technologies: the potential mHealth approach

**DOI:** 10.3389/fpsyg.2014.00559

**Published:** 2014-06-10

**Authors:** Gianluca Castelnuovo, Gian Mauro Manzoni, Giada Pietrabissa, Stefania Corti, Emanuele Maria Giusti, Enrico Molinari, Susan Simpson

**Affiliations:** ^1^Department of Psychology, Catholic University of MilanMilan, Italy; ^2^Istituto Auxologico Italiano Istituto di Ricovero e Cura a Carattere Scientifico, Psychology Research LaboratoryOspedale San Giuseppe, Verbania, Italy; ^3^Faculty of Psychology, eCampus UniversityComo, Italy; ^4^Department of Psychology, University of BergamoBergamo, Italy; ^5^School of Psychology, Social Work and Social Policy, University of South AustraliaAdelaide, SA, Australia

**Keywords:** obesity, rehabilitation, telemedicine, telecare, mHealth, clinical psychology, health psychology, psychotherapy

## Abstract

Obesity is currently an important public health problem of epidemic proportions (globesity). Inpatient rehabilitation interventions that aim at improving weight-loss, reducing obesity-related complications and changing dysfunctional behaviors, should ideally be carried out in a multidisciplinary context with a clinical team composed of psychologists, dieticians, psychiatrists, endocrinologists, nutritionists, physiotherapists, etc. Long-term outpatient multidisciplinary treatments are likely to constitute an essential aspect of rehabilitation. Internet-based technologies can improve long-term obesity rehabilitation within a collaborative approach by enhancing the steps specified by psychological and medical treatment protocols. These outcomes may be augmented further by the mHealth approach, through creating new treatment delivery methods to increase compliance and engagement. mHealth (m-health, mobile health) can be defined as the practice of medicine and public health, supported by mobile communication devices for health services and information. mHealth applications which can be implemented in weight loss protocols and obesity rehabilitation are discussed, taking into account future research directions in this promising area.

## INTRODUCTION

Obesity, defined as a body mass index (BMI) of 30 kg/m^2^ or higher, is today considered an important public health problem and epidemic (globesity; Gutierrez-Fisac et al., 2006; Capodaglio and Liuzzi, 2013). Obesity is also associated with early death and universally recognized as a risk factor for many health complications and disabilities such as cardiovascular diseases, osteoarthritis, hypertension, dyslipidemia, hypercholesterolemia, Type-2 diabetes and cancer (Flegal et al., 2005; Whitlock et al., 2009; Capodaglio et al., 2010, 2011; Castelnuovo et al., 2010; Capodaglio and Liuzzi, 2013).

There is general consensus among professionals that the etiology of obesity is multifactorial with interaction between genetic, individual, and environmental factors (Marcus and Wildes, 2009). Even if genetics plays an important role in the etiology of obesity, according to Dombrowski, “Behavioral factors, i.e., poor diet and physical inactivity are among the main proximal causes linked to obesity... obesity-related morbidity... and mortality...” (p. 7, 2012). Moreover social, psychological, and psychopathological variables are clear determinants in the development and treatment of obesity (Davin and Taylor, 2009). For example, epidemiologic investigations have revealed significant correlations between obesity and eating disorders, mood disorders, anxiety disorders, and personality disorders (Hudson et al., 2007; Pickering et al., 2007; Petry et al., 2008; Scott et al., 2008; Villa et al., 2009; Manzoni et al., 2010).

In the context of in-patient rehabilitation, interventions aimed at improving weight-loss, reducing obesity-related complications and changing dysfunctional behaviors should be typically carried out in a multidisciplinary context (with a clinical team composed by, dieticians, endocrinologists or nutritionists, physiotherapists, psychiatrists, psychologists surgeons, etc.). There may be additional benefit from the inclusion of specific instructions for changing diet and self-monitoring dietary intake, whilst providing guidance and support in maintaining goals initially achieved, anticipating possible future relapses and learning strategies to cope with difficult moments or situations (Capodaglio et al., 2010, 2013a,b; Manzoni et al., 2010, 2011b; Dombrowski et al., 2011, 2012; Capodaglio and Liuzzi, 2013). A range of psychological approaches may be suitable for the in-patient treatment of obesity, such as behavioral, cognitive-behavioral, interpersonal, systemic-strategic, psychodynamic, schema etc.; Shaw et al., 2005; Castelnuovo, 2010a,b). Among these different approaches, cognitive-behavior therapy (CBT) represents the gold standard for the treatment of obesity, focusing on dysfunctional behaviors, cognitive processes, unrealistic weight goals and body image perceptions (Murphy et al., 2010). The combination of psychological therapy and diet/exercise plans, leads to better weight loss outcomes than diet/exercise interventions alone. Psychological and behavioral treatments generally include out-patient follow-up sessions which facilitate ongoing assessment an guidance in a range of areas. This may include determining clients’ ability to self-monitor (for example, using diaries), assistance with stimulus control (for example, restricting quantities of food) and behavioral modification strategies (for example, chewing slowly, taking time to taste and enjoy food, and increasing awareness of the pleasure associated with taste; Wing, 2002; Foster et al., 2005; Swencionis and Rendell, 2012).

In a multidisciplinary obesity rehabilitation approach, it is important to underline that treatment could involve non-pharmacological, pharmacological and surgical methods. Nowadays functional anti-obesity drugs are partially indicated for those who are obese with one or more weight-related comorbid conditions (Rueda-Clausen et al., 2013; Kushner, 2014; Patham et al., 2014). Moreover additional interventions could be necessary: bariatric surgery can be an effective approach for weight loss and comorbidity reduction, taking into account that surgery can generate considerable risks and can be advised only to selected patients (Sandoval, 2011; Simpson et al., 2011; Henry et al., 2013; Kushner, 2014).

## OBESITY REHABILITATION NEEDS OUT-PATIENT LONG-TERM STEPS

Recent studies have underlined the role of the neural reward system in the development and maintenance of obesity: “dysfunction of brain reward circuitry in response to food cues may predispose some individuals to obesity via an increased likelihood of overeating, particularly excessive consumption of palatable foods” (p. 744, Marcus and Wildes, 2009). Thus some kinds of obesity may be considered an expression of food “addiction,” problem that typically requires a long-term treatment (Wang et al., 2001, 2002, 2004, 2009; Gearhardt et al., 2009, 2011a,b,c,d, 2012, 2013; Gearhardt and Corbin, 2011; Gearhardt and Brownell, 2013).

Moreover binge eating disorder (BED) is typically connected with obesity (American Psychiatric Association, 2000; Hill, 2005; Berkowitz and Fabricatore, 2011; Gearhardt et al., 2011c; Wilson, 2011; Schag et al., 2013; Faulconbridge and Bechtel, 2014), even if not occurring exclusively in conjunction with overweight conditions. According to Hill (2005, p. 27), “it is apparent that BED is more common in the obese than in normal-weight individuals. In US weight loss clinics, 20–40% of patients are reported to have BED, although the use of a strict diagnostic interview reduces this to well below 20%. In community samples, BED is much less common, apparent in 1–3% of respondents. Overall, the prevalence of BED in any group increases with increasing obesity.” Higher levels of psychological distress and self-esteem problems are associated with obesity with BED. Typically obesity with BED requires a longer term treatment in comparison with simple obesity (Hill, 2005).

Moreover, while from a clinician’s point of view a 10% weight loss is generally considered an important success due to a significant reduction in comorbidities and complications, patients typically have higher expectations, perceiving that a good result constitutes a minimal 30% body weight reduction. Thus, establishing genuine and achievable expectations of weight loss represents an important challenge for the management of obesity (Foster et al., 1997; Jeffery et al., 1998; O’Neil et al., 2000; Wadden et al., 2000). Ongoing psychological support is required to assist patients in developing more realistic weight loss outcomes as well as in motivating them to follow rehabilitation programs (Foster et al., 2005; Nonas and Foster, 2005). Although moderate weight loss (5–10% of initial weight) can lead to positive psychological changes, such as improvements in body satisfaction, self-esteem and mood (Hill, 2005), these findings tend to be associated with short-term studies. Typically, a long-term psychotherapeutic treatment is required in order to sustain realistic weight loss expectations and motivation to change (Hill, 2005).

Taking into account previous considerations, if we consider obesity to be a chronic form of food addiction, which may in some cases be accompanied by BED and unrealistic expectations of weight loss, long-term multidisciplinary treatment is likely to lead to optimal outcomes both across in-patient and out-patient settings.

Also, a collaborative approach, defined as a “strategy or set of strategies to help patients achieve and/or maintain a healthy weight that involve collaboration among healthcare professionals in at least two different disciplines (e.g., physicians and dieticians) for the delivery of weight management interventions” (p. 1190, Rao et al., 2011) is required. Thus strategies based on central planning, grounded in a “chronic care model” logic, tend to obtain better results, although at this early stage only a limited number of articles have reported real and practical collaborative experiences, largely in out-patient settings (Rao et al., 2011).

## NEW TECHNOLOGIES FOR OUT-PATIENT OBESITY REHABILITATION: THE TECNOB PROJECT

Internet-based technologies provide patients with continuous and remote psychological, medical and nutritional support and education in order to enhance motivation, compliance and engagement, thereby maximizing the benefits of collaborative outpatient rehabilitation programs (Castelnuovo et al., 2003, 2010, 2011a,b,c,d; Riva et al., 2006; Castelnuovo and Simpson, 2011; Manzoni et al., 2011a; Rao et al., 2011; Simpson and Slowey, 2011).

Moreover the use of telemonitoring and telecare approaches that ensure continuity of care in out-patient settings can contribute to a significant cost reduction in the management of obesity and other chronic pathologies (Ekeland et al., 2010, 2011; Manzoni et al., 2011a).

One such pioneering example of a collaborative approach is the TECNOB Project (TEChNology for OBesity; Castelnuovo et al., 2003, 2010, 2011a,b; Castelnuovo, 2007). It runs for a total duration of 13 months and consists of two consecutive phases: in-patient (1 month) and out-patient (the following 12 months). The clinician-patient relationship is considered a highly significant agent and vehicle for change. After discharge, out-patients begin to experience a sense of autonomy and competence as they continue the change process they have begun to develop during the in-patient phase, whilst learning to face a range of resistances and barriers. Through the use of videoconferencing out-patients are supported by the clinicians who worked with them during the in-hospital phase, through exploring resistances and impediments they experience and finding functional and healthy coping mechanisms. Furthermore, out-patients are helped to experience a sense of mastery as they become proficient at attaining healthy behavioral changes.

Other positive experiences are well reported and described in (Bacigalupo et al., 2013), where the common components of the Internet-based clinical protocols are few (self-monitoring related to weight and physical activity and automatic-professional feedback to participants), whereas the intervention programs varied significantly in many details and features (Bacigalupo et al., 2013).

## NEW TECHNOLOGIES FOR OUT-PATIENT OBESITY REHABILITATION: THE mHealth SCENARIO

Internet-based tools can provide promising results in enhancing weight reduction among obese patients but further studies are required in order to determine its long-term efficacy and effectiveness across clinical, organizational, and economic perspectives. (Manzoni et al., 2008, 2011a; Khaylis et al., 2010; Rao et al., 2011).

Until now the published data has not supported the competitive use of Internet interventions for weight loss and maintenance in out-patient settings. In spite of this lack of literature, promising clinical reports have been published about the usefulness of mobile phone devices in promoting healthy habits and weight loss attitudes (Rao et al., 2011; Park and Kim, 2012; Pellegrini et al., 2012; Schiel et al., 2012; Bacigalupo et al., 2013; Hebden et al., 2013; Rodrigues et al., 2013; Schoffman et al., 2013; Sharifi et al., 2013; Shaw et al., 2013).

Moreover, no unequivocal data have been collected about real costs of telemedicine. Certainly it could reduce travel time, hospital admissions and indirect costs to service users and their social networks (Ekeland et al., 2010, 2011; Khaylis et al., 2010; Hilty et al., 2013). The potential for technical problems as well as skepticism or reticence from patients, caregivers, nurses, and physicians may limit the spread of e-health solutions (e.g., Rees and Stone, 2005). The mHealth approach has the potential to make contributions not only in adult obesity (Tufano and Karras, 2005; Burke et al., 2012) but also in pediatric obesity (Jensen et al., 2012; Turner-McGrievy et al., 2013), thereby creating new treatment delivery methods that could increase participation, compliance and engagement (Graffigna et al., 2013a,b). About pediatric obesity, Cohen et al. (2012) in a recent review noted that telemedicine could be a promising approach to pediatric weight management, particularly for families in rural contexts with limited access to traditional treatments, even if many doubts are present again, particularly what treatment components (psychological support, lifestyle modification, nutritional education, medical prescription, etc.) could functionally fit into the e-health settings.

According to Eysenbach (2001, p. 1), e-health could be defined as “an emerging field in the intersection of medical informatics, public health and business, referring to health services and information delivered or enhanced through the Internet and related technologies. In a broader sense, the term characterizes not only a technical development, but also a state-of-mind, a way of thinking, an attitude, and a commitment for networked, global thinking, to improve health care locally, regionally, and worldwide by using information and communication technology.” e-health is characterized by the presence of 10 features: efficiency, enhanced quality of care, evidence-based approach, empowerment of consumers and patients, encouragement of a new relationship between the patient and health professional, on-line education of physicians, information and communication exchange, extension of the health care scope beyond its conventional boundaries (in both geographical and conceptual sense), ethics and equity (Eysenbach, 2001). mHealth (m-health, mhealth, mobile health) could be defined as the practice of medicine and public health, supported by mobile communication devices, such as mobile phones, tablet computers, and PDAs, for health services and information (Riper et al., 2010; Eysenbach, 2011; Cipresso et al., 2012; Whittaker, 2012; Fiordelli et al., 2013). mHealth applications have also been implemented with promising applications and results in weight loss protocols and obesity rehabilitation (Chomutare et al., 2011; Burke et al., 2012; Cafazzo et al., 2012; Fiordelli et al., 2013; Martinez-Perez et al., 2013; Turner-McGrievy et al., 2013).

## FIVE PSYCHOLOGICAL COMPONENTS TO BE CONSIDERED IN mHealth WEIGHT-LOSS APPLICATIONS

According to Khaylis et al. (2010, p. 932–936) five psychological components need to be considered for technology-based and mHealth-based obesity rehabilitation in order to facilitate weight-loss.

### SELF-MONITORING

Self-monitoring refers to the process in which individuals regulate and keep track of their own behaviors. Technology can simplify the monitoring process, recording one’s progress of food intake and physical activity using online devices. The reason these technologies are likely to be effective is because portable body monitors, pedometers, and handheld PDAs are mobile and, therefore, can be easily used, resulting in continuous self-monitoring. Also, these devices are more convenient for individuals without access to a high-speed Internet connection.

### COUNSELOR FEEDBACK AND COMMUNICATION

Feedback from a counselor regarding goals, progress, and results can encourage, motivate, and assist patients in successfully completing a weight-loss program. A functional approach is to provide online weight-loss interventions with brief weekly or monthly counselor or psychologist visits. Participants typically submit their weekly food and exercise journals online, receiving personalized feedback, reinforcement, and recommendations from a counselor over e-mail.

### SOCIAL SUPPORT

A group treatment format is typically preferred for behavioral weight-loss interventions. Not only does it constitute a cost-effective method for delivering treatment to a larger number of people, but it also enhances social support, an important facilitator of behavioral change. Group support can foster motivation, encouragement, and commonality. To facilitate communication among participants electronic message boards, forums, “real time” chat rooms or online meetings represent useful tools.

### STRUCTURED PROGRAM

Technology-based weight-loss programs incorporate principles of behavior therapy and change. They consist of structured weekly lessons on various topics, including nutrition, exercise, stimulus control, self-regulation strategies, goal-setting.

### INDIVIDUALLY TAILORED PROGRAM

Interventions specifically designed around individual’s goals typically record higher rates of adherence and weight loss. In one report, participants were required to meet with a health coach and select four high-priority behavioral change goals, before being monitored through a behavioral skills training program. In another study real-time SMS text messages were delivered to each patient, as a direct challenge to pre-identified barriers to exercise.

## FUTURE DIRECTIONS IN CLINICAL PSYCHOLOGY FOR OBESITY REHABILITATION

Potential benefits of mobile monitoring methods for behavioral weight loss protocols appear clear (Turner-McGrievy et al., 2013). However, “future studies should examine ways to predict which self-monitoring method works best for an individual to increase adherence” (Turner-McGrievy et al., 2013, p. 513). There is a critical need for scientific research to evaluate the specific outcomes of collaborative approaches for weight management that utilize Internet and mobile based tools.

mHealth approach could help clinicians by motivating patients in remote settings to develop healthier lifestyles (Castelnuovo, 2010a; Pietrabissa et al., 2012), to accept more intrusive medical treatments (such as drugs and weight-loss surgery), to cope with chronic conditions and to reduce complications (such as Type-2 Diabetes, hypertension and cardiovascular disease; Nguyen and Lau, 2012).

Moreover clinicians should adhere to good professional practice protocols in technological settings: “discussions of weight should be performed in a non-judgmental, respectful, and unhurried manner” (p. 1200, Rao et al., 2011), “readiness and self-efficacy to change behaviors should be assessed before weight loss strategies are initiated, and this information should be factored into decisions about what type of approach to use” (p. 1200, 2011), and collaborative approaches involving physicians, psychologists, nurses, and other clinicians need to be considered by utilizing consistent planning and training modalities.

Future directions in obesity and weight-reduction research have been provided by Rao et al. (2011, p. 1200):

(1) “There is a need for larger studies, both those that include technologically based interventions and those that do not, that enroll a diverse spectrum of overweight and obese patients in terms of sex, race, and socioeconomic status. Latino subjects and men, in particular, are underrepresented in obesity studies to date. There is also a need to investigate the specific features of technologically based interventions (e.g., content, format, device) that make such interventions successful in promoting weight loss.
(2) Because attrition rates from technology-based studies are very high, there is a need to develop effective strategies to keep patients engaged in using technology tools for the long-term.
(3) Further evaluation of collaborative approaches (e.g., approaches involving centralized planning, approaches involving nurses in intervention delivery) in general is needed. In particular, larger studies of longer duration are needed to evaluate the effectiveness of the chronic care model as a framework for weight management interventions.
(4) Use of electronic health records is increasing, and there is a need to explore the use of these valuable tools, not only for identification and assessment of obesity but also for the delivery of obesity interventions.”
  Whittaker (2012, p. 6) provides some methodological suggestions for future research in this issue: “evaluations of effectiveness and usability are required and should be made publicly available. Where evaluation is planned during the development stage, data collection can be built in as an integral part of the program. The ideal of randomized controlled trials will still be necessary in some contexts. In these cases, careful consideration should be given to the appropriate comparator to ensure the right question is being answered. For example, what is usual care for this target audience? Can we measure an improvement in access as an outcome? Other research methods will be more appropriate in other circumstances, such as adaptive trials to allow the intervention to develop and improve as part of the research; observational trials and qualitative research methods to detect unintended consequences and changes to workflow; and qualitative studies to test acceptability. Evaluating effectiveness and usability is also possible while implementing a system, for example, with novel designs such as the stepped wedge cluster randomized trial, and particularly where there is little likelihood of harm.”

To conclude, further studies should investigate both possible advantages and applications of Internet and mHealth technologies in the treatment of obesity. In spite of promising preliminary reports, the evidence-base for the effectiveness of mHealth applications is meager and it remains too early to be able to recommend it for use in clinical settings.

## Conflict of Interest Statement

The authors declare that the research was conducted in the absence of any commercial or financial relationships that could be construed as a potential conflict of interest.
